# Extensive Post‐Zoster Keloid Formation in an Immunocompetent Individual: An Unusual Wolf's Isotopic Response

**DOI:** 10.1002/ccr3.70826

**Published:** 2025-08-30

**Authors:** Mahesh Mathur, Sumit Paudel, Nabita Bhattarai, Sandhya Regmi, Sambidha Karki

**Affiliations:** ^1^ Department of Dermatology College of Medical Sciences Bharatpur Nepal

**Keywords:** herpes zoster, immunocompetence, keloid, post zoster scarring, Wolf's isotopic response

## Abstract

Extensive keloid formation may develop at the site of healed herpes zoster lesions as a rare Wolf's isotopic response, even in immunocompetent individuals without a medical history of keloid, highlighting the importance of early antiviral therapy to minimize post‐herpetic complications.

**Abbreviations**
HIV: human immunodeficiency virus
HZ: herpes zoster
VZV: varicella zoster virus
WIR: Wolf's isotopic response

## 1 Introduction

Herpes zoster (HZ) is a localized viral neurocutaneous disease resulting from the reactivation of dormant varicella zoster virus (VZV) within the dorsal root ganglion [1]. Postherpetic Wolf's isotopic response (WIR) is a phenomenon when an unrelated skin condition appears in an area previously affected by HZ. HZ is the most common condition preceding this response. However, keloid after HZ lesions is rare, typically occurring in individuals predisposed to keloid development or a weakened immune system [2]. We hereby report a case of unusually extensive keloid at the site of prior HZ in an immunocompetent patient with no keloidal tendency.

## 2 Case History

**FIGURE 1:**
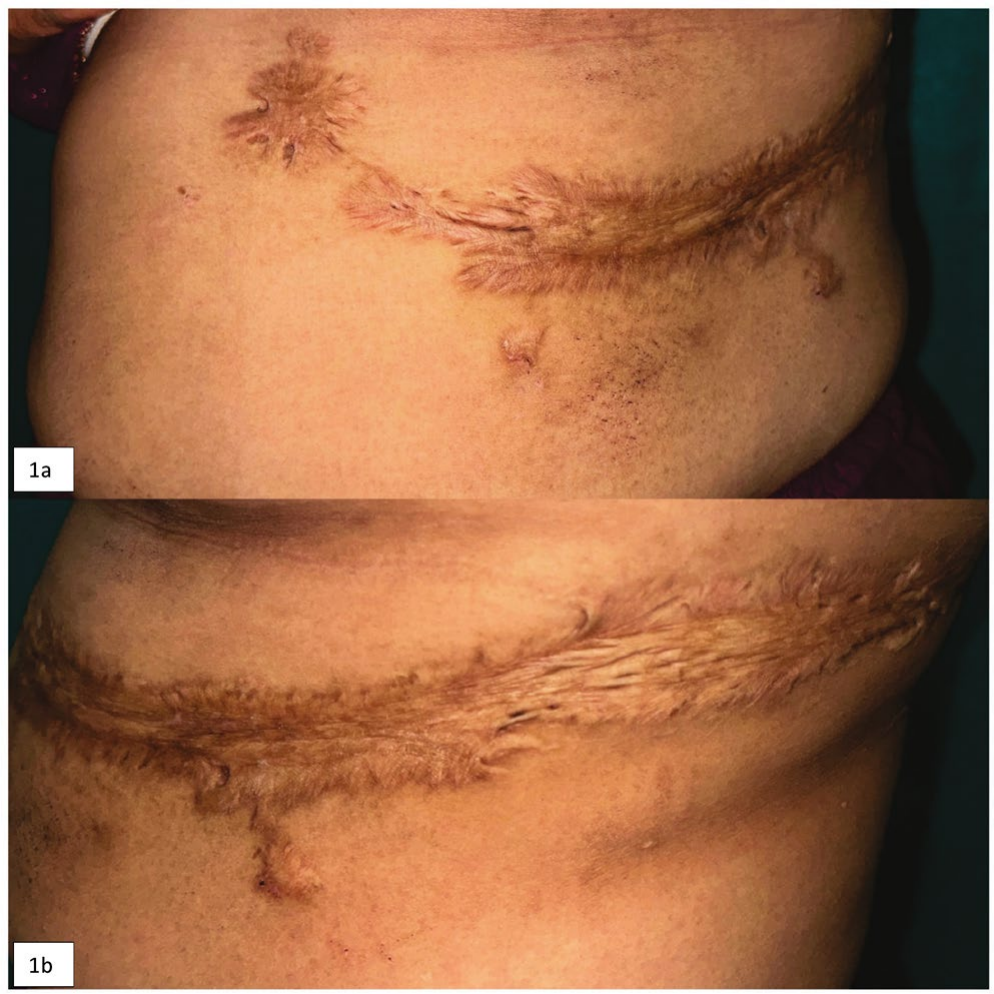
Keloid scar on anterior (a) and lateral (b) aspect of the left side of the abdomen at the site of previous Herpes Zoster lesion, corresponding to dermatome left T6 and T7 extending beyond the original wound margin with “claw like” extensions.

A 45‐year‐old female presented with a progressively expanding, irregular, yellowish‐brown colored elevated lesion on her abdomen and mid‐trunk on the left side over 11 months. (Figure 1a,b) She had an episode of HZ at the same site 1 year back. She received symptomatic treatment only. The lesion healed with hyperpigmentation without deep necrosis or secondary infection. One month after the lesion, she started developing prominent, expanding scars with characteristic keloidal features. She had no personal or family history of keloid.

On examination, ill‐defined, firm, rubbery, flesh‐colored to hyperpigmented plaques and nodules following a zosteriform pattern in mid‐trunk and abdomen on the left side, corresponding to dermatome left T6 and T7 were present. (Figure 1a,b).

## 3 DIFFERENTIAL DIAGNOSIS: (Table 1)

**TABLE 1:** Differential diagnosis of Post Zoster Keloid.

Differential diagnosis	Differentiating point from Post Zoster Keloid
Hypertrophic scar	Confined to wound margin, spontaneously regress with time, fine wavy collagen bundles parallel to epidermis
2 Dermatofibrosarcoma Protuberans	Expanding keloidal plaque with nodules, red blue to brown color, spindle‐shaped cells arranged in a “storiform” pattern.
3 Scar Sarcoidiosis	Dusky red to violaceous indurated plaques or nodules with progressive enlargement over preexisting scars, typical sarcoid (non caseating/naked) granuloma
4 Keloidal Morphea	Firm, sclerotic indurated, keloid like plaque or nodule with liliac border occurring in absence of prior trauma or injury, dermal sclerosis and diminished or absent adnexal structures.

1. Hypertrophic scar
2. Dermatofibrosarcoma Protuberans
3. Scar sarcoidosis
4. Keloidal morphea

## 4 Results

**FIGURE 2:**
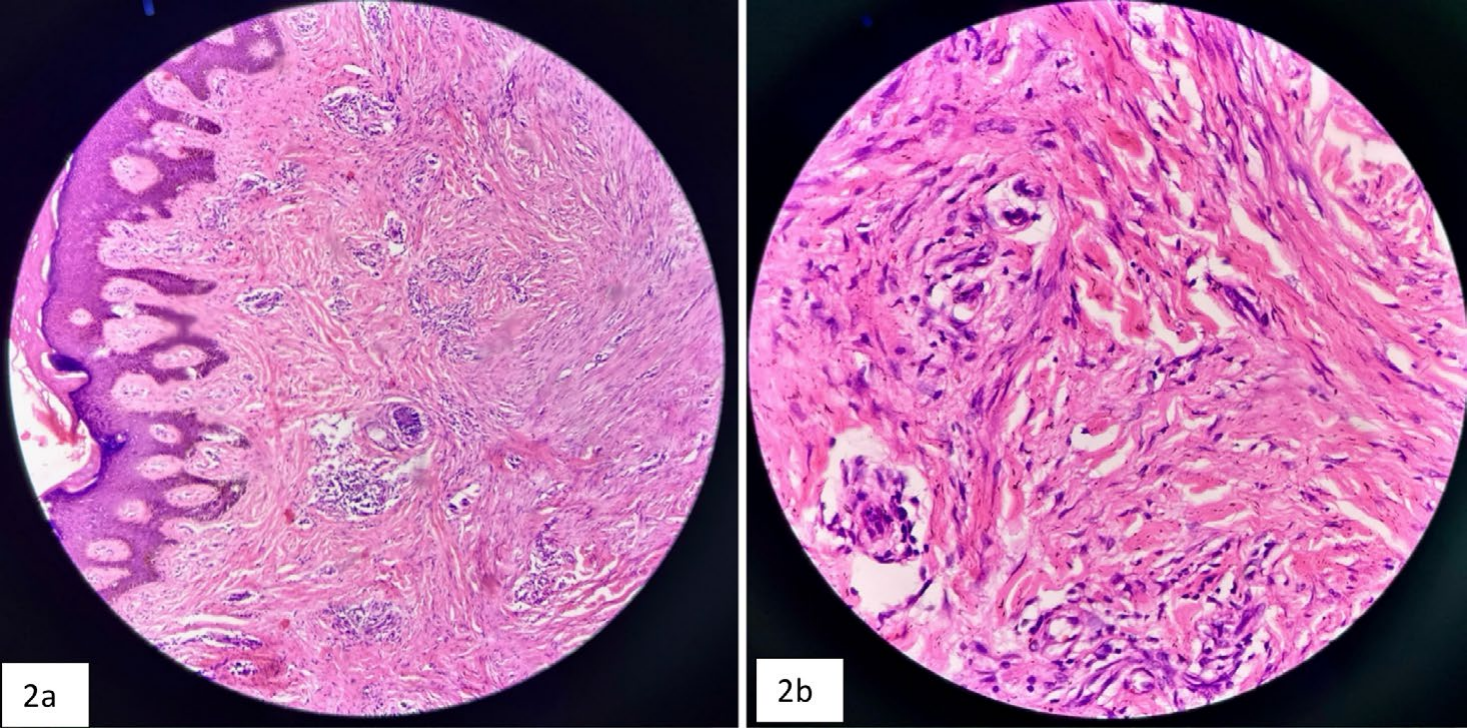
(a, b) (40×, 100× respectively): Histopathological finding of Post Zoster Keloid. Characteristic “keloidal collagen” composed of broad, homogeneous, brightly eosinophilic bands of hyalinized thickened collagen bundles, haphazardly arranged in the reticular dermis sparing the papillary dermis. Presence of fibroblasts between or around the collagen bands.

Histopathological examination revealed thick, hyalinized, eosinophilic collagen bundles termed “Keloidal collagen” arranged haphazardly in the dermis with minimal inflammation and sparse fibroblasts, consistent with findings of a keloid. (Figure 2a,b) Routine blood investigations were normal. Screening for human immunodeficiency virus (HIV) was negative. She was counseled about the nature and prognosis of the disease.

## 5 Discussion

Keloids develop as a result of abnormal wound healing, influenced by both environmental and genetic factors, and are more frequently seen in people with darker skin tones [3]. Overactive fibroblasts produce excessive collagen and growth factors, leading to scars that extend beyond the original wound [2].

Described initially in 1955, WIR refers to the occurrence of a new, unrelated skin disorder at the location of a previously resolved dermatosis [2]. The pathogenesis of WIR following HZ involves viral, immune, vascular, and neural mechanisms [1]. VZV can disrupt local immunity by impairing dendritic cell function, altering microcirculation, and causing collagen rearrangement [2]. The “immunocompromised skin” theory suggests prior injury or infection creates localized immune dysregulation with increasing susceptibility to new lesions [4].

Granulomatous, lichenoid reactions, and skin tumors are commonly reported isotopic responses following HZ [2, 4]. Only a few cases of keloid scar formation following HZ have been reported in the literature, mostly small, localized keloids occurring in individuals with underlying comorbidities or immunocompromised states, such as HIV infection [2] or a history of renal transplantation [5].

Extensive cosmetically significant keloid in an immunocompetent patient without comorbidities, as seen in our case, is an unusual and severe manifestation of WIR. We hypothesize that the absence of antiviral treatment during the acute HZ episode may have prolonged local inflammation leading to dysregulated immune signaling, fibroblast activation, and subsequent keloid formation. This suggests that localized immune dysregulation can trigger keloid formation without systemic immune suppression or any genetic predisposition for keloid [4].

Herpes zoster is a common infectious condition. However, keloid formation is an extremely rare but well‐documented isotopic response of HZ [4]. Keloids are aesthetically unpleasant and significantly impact the patient's quality of life [2]. Localized immune dysregulation at a healed HZ lesion can lead to keloid formation. Viral medication early in the onset of the disease may restrict the inflammation, eventually preventing the activation of fibroblasts and keloid formation [4]. Furthermore, early treatment of keloid is essential to improve cosmetic and functional outcomes. This case adds to the limited literature on post‐herpetic keloid formation and heightened clinical awareness of atypical sequelae following HZ. It also highlights the importance of considering key differential diagnoses to facilitate prompt and accurate management.

## Author Contributions

**Mahesh Mathur:** conceptualization, formal analysis, supervision, validation, visualization, writing – review and editing. **Sumit Paudel:** formal analysis, supervision, writing – review and editing. **Nabita Bhattarai:** data curation, writing – review and editing. **Sandhya Regmi:** data curation, writing – review and editing. **Sambidha Karki:** conceptualization, formal analysis, resources, supervision, validation, visualization, writing – original draft.

## Disclosure

Prior publication: This material has not been published previously.

## Ethics Statement

The patients in the manuscript have given written informed consent to the publication of their case details.

## Consent

Written informed consent was taken from all the patients to publish this case report in accordance with the journal's patient consent policy.

## Conflicts of Interest

The authors declare no conflicts of interest.

## Data Availability

The data that support the findings of this study are available from the corresponding author upon reasonable request.
